# Variation in the Outcome of Norepinephrine-Dependent Septic Patients After the Institution of a *Patient-Tailored Therapy* Protocol in an Italian Intensive Care Unit: Retrospective Observational Study

**DOI:** 10.3389/fmed.2020.592282

**Published:** 2020-11-05

**Authors:** Erika Casarotta, Elisa Damiani, Roberta Domizi, Andrea Carsetti, Claudia Scorcella, Erica Adrario, Sandra Bolognini, Domenico Di Falco, Simona Pantanetti, Sara Vannicola, Agnese Damia Paciarini, Abele Donati

**Affiliations:** ^1^Anesthesia and Intensive Care Unit, Department of Biomedical Sciences and Public Health, Università Politecnica Delle Marche, Ancona, Italy; ^2^Anesthesia and Intensive Care Unit, Azienda Ospedaliera Universitaria “Ospedali Riuniti” of Ancona, Ancona, Italy

**Keywords:** sepsis, septic shock, patient-tailored therapy, outcome, mortality

## Abstract

**Objective:** To evaluate the outcome of patients with septic shock after the institution of a *patient tailored therapy* protocol in our Intensive Care Unit (ICU).

**Methods:** Single-center retrospective observational study including 100 consecutive septic patients (≥ 16 years) requiring norepinephrine infusion, admitted to our ICU between 2018 and 2019 after the institution of a *patient-tailored therapy* protocol, compared with a historical control group of 100 patients admitted between 2010 and 2013 (*historical controls*). The *patient-tailored therapy* protocol included the use of IgM-enriched immunoglobulins for patients with low plasma IgM levels, blood purification strategies for patients with high plasma levels of cytokines or endotoxin, albumin correction and modulation of vasoactive agents. Clinical and therapeutic parameters were noted at the time of initiation of norepinephrine infusion and for the 1st 24 h. The primary outcome was ICU mortality.

**Results:** ICU-mortality was lower in the patient-tailored therapy cohort as compared to historical controls (32 vs. 57%, *p* < 0.001). Patient-tailored therapy was associated with a lower risk of ICU-mortality even after adjusting for the main clinical severity indices (adjusted odds ratio 0.331 [95% confidence interval 0.166–0.658], *p* = 0.002). After propensity score matching, 48 patients in historical control group and 48 patients in the patient-tailored therapy cohort with similar general characteristics were selected. ICU-mortality was lower in the patient-tailored therapy matched subgroup as compared to historical controls (40 vs. 60%, *p* = 0.037).

**Conclusions:** An individualized therapeutic approach in septic patients may be associated with a survival benefit. However, the use of an historical control group of patients admitted between 2010 and 2013 may introduce substantial bias. Further adequately designed studies are needed to demonstrate the impact of *patient-tailored therapy* on outcome.

## Introduction

Sepsis is defined as life-threatening organ dysfunction caused by a dysregulated host response to infection (1). Septic patients are a heterogeneous population (2), not only because of the pathophysiological complexity underlying this syndrome, but also because of the different basal characteristics of individual patient: for this reason, it is difficult to find a single therapy that may be effective for everyone (3, 4). For each patient, the therapeutic strategy must be based on the underlying physiological reserve, pre-existing comorbidities and organ dysfunction severity (2), with a *patient tailored therapy* approach. For example, recent evidence suggests that hemodynamic management must be optimized in each patient based on dynamic evaluation of clinical and laboratory parameters, indicative of organ perfusion (5), and on the previous clinical history. A multicentre trial showed that a higher blood pressure target may be required in patients with a history of arterial hypertension (6), suggesting that an individualized approach may be preferable. Similarly, different immunomodulation treatments, including intravenous immunoglobulins or extracorporeal blood purification techniques, taken individually, did not show a clear positive correlation with outcome (5), but a more careful patient selection (based on immunoglobulin and cytokine levels) may be necessary to better show an impact on survival.

Starting from 2018, a *patient tailored therapy* protocol has been applied in our Intensive Care Unit (ICU) for the management of sepsis and septic shock. This protocol involves the use of adjunctive therapies, modulated according to the patient's characteristics, including intravenous immunoglobulins, extracorporeal removal strategies and hemodynamic support.

The primary goal of this study was to evaluate the outcome of patients with sepsis/septic shock admitted to our ICU after the institution of this *patient tailored therapy* protocol, in comparison to an historical control group of patients who were treated in an earlier period. Secondary endpoints were hospital mortality, ICU length of stay, maximum dose of norepinephrine and total fluid intake in the 1st 24 h after sepsis diagnosis, fluid balance at 24 h.

## Materials and Methods

This single-center retrospective observational study was conducted in the Intensive Care Unit of Azienda Ospedaliero-Universitaria “Ospedali Riuniti” of Ancona, in Italy. The study protocol was approved by the local ethic committee (Comitato Etico Regionale delle Marche) before the data were accessed. The patient records were de-identified before the data were accessed, and the data were analyzed anonymously. Written informed consent was not necessary due to the retrospective nature of the study and because the data were analyzed anonymously. We included 100 consecutive patients (≥ 16 years old) with sepsis (1) requiring norepinephrine infusion, admitted to our ICU between July 2018 and September 2019 (patient-tailored therapy group). Clinical records were reviewed in order to select those patients who required an infusion of norepinephrine for persistent sepsis-induced hypotension. Persistent hypotension was defined by a systolic arterial pressure below 90 mmHg, or mean arterial pressure lower than 60 mmHg, or a reduction in systolic blood pressure of more than 40 mmHg from baseline, despite adequate volume resuscitation, in the absence of other causes of hypotension, requiring the infusion of vasopressors (7). According to current and previous guidelines of the *Surviving Sepsis Campaign* (8, 9), adequate volume resuscitation was defined by the absence of hemodynamic improvement after a fluid challenge either based on dynamic or static hemodynamic variables. Septic shock was defined as an acute circulatory failure characterized by persistent hypotension despite adequate fluid resuscitation, requiring vasopressor infusion, with arterial lactate levels > 2 mmol/l (1). As historical controls, we used a group of 100 consecutive patients with norepinephrine-dependent sepsis admitted to our ICU between December 2010 and January 2013, who had been already enrolled for a previous retrospective study (10). That multicentre study was aimed to test the association between tachycardia and mortality and involved adult patients with a diagnosis of septic shock requiring norepinephrine as the first-line vasopressor despite adequate volume resuscitation (10), according the 2001 definitions (7). Exclusion criteria were in both studies: age < 16 years; duration of norepinephrine infusion < 6 h or survival time <6 h after the introduction of norepinephrine (10).

### Patient-Tailored Therapy Protocol

**Figure 1:**
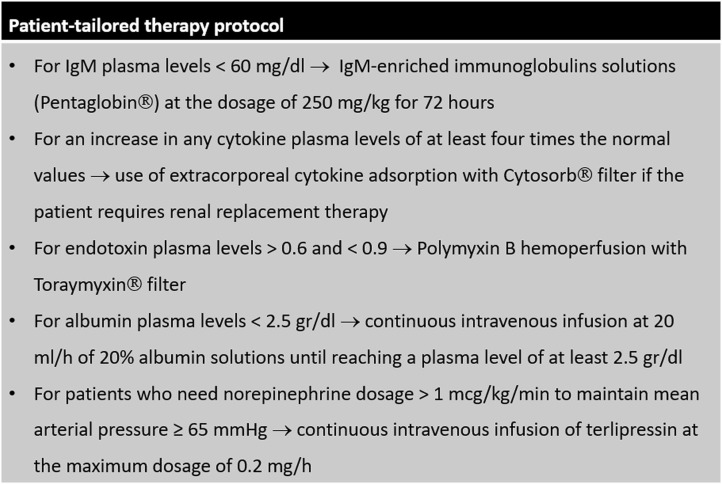
Patient-tailored therapy protocol.

Starting from 2018, a *patient-tailored therapy* protocol for the management of sepsis has been systematically applied in our ICU. This protocol is shown in details in Figure 1. After diagnosis of sepsis, dosage of plasma levels of immunoglobulins, cytokines (interleukin (IL) 1-beta, IL-6, IL-8, IL-10, and tumor necrosis factor (TNF) alpha), endotoxin and albumin is performed in all patients. When plasma levels of IgM are low, septic patients receive IgM-enriched immunoglobulins solutions; when plasma levels of cytokines or endotoxin are high, extracorporeal removal strategies are applied; albumin replacement is indicated for patients with low plasma levels. To achieve a value of mean arterial pressure ≥ 65 mmHg, in patients who would require high norepinephrine dosage, terlipressin is added. All other therapies were based on the indications of the *Surviving Sepsis Campaign* (8). Before 2018, all septic patients were treated according to current guidelines and based on the indications of the attending physician, without a protocolized approach.

### Data Collection

Data collection was performed by different researchers for the historical and the interventional cohorts, using an identical data extraction form. Data extracted for the 2018–2019 cohort were double-checked by the investigator who had collected data for the 2010–2013 cohort, in order to make sure that the same criteria had been applied for selecting patients and that those who had received norepinephrine for hypotension due to other causes were excluded. For each patient, source of infection, pathogen involved, multidrug resistance [defined as: Gram positive – methicillin-resistant; Gram negative – resistant to at least 3 antibiotic classes; Candida – resistant to fluconazole (10)] and state of immunosuppression [defined as: neutrophils <500 cells/mm^3^ or chemotherapy administration in the previous 2 weeks or prednisone 20 mg/day or equivalent (10)] were noted. The *Sequential Organ Failure Assessment* (SOFA) score, *Simplified Acute Physiology Score* (SAPS II), and the *Acute Physiology and Chronic Health Evaluation* (APACHE) score of the 1st 24 h after ICU admission were calculated. At the time of initiation of norepinephrine infusion and for the 1st 24 h the following parameters were registered: body temperature, heart rate, heart rhythm, systolic arterial pressure, diastolic arterial pressure, mean arterial pressure, norepinephrine and other vasoactive drugs dosage, cardiac output (if available), sedative drugs dosage, main mechanical ventilation parameters, need for renal replacement therapy. We calculated SOFA score at the time of initiation of norepinephrine and for the 1st 24 h and the fluid balance for the 1st 24 h. The primary outcome was ICU mortality. We also noted in-hospital mortality, ICU length of stay, hospital length of stay, cause of death.

### Sample Size

In the previous retrospective study 100 patients diagnosed with septic shock (7) were enrolled. These patients were used as historical controls. We arbitrarily decided to collect data of an equal number of patients admitted to our ICU after the institution of this *patient tailored therapy* protocol.

### Statistical Analysis

This was performed using GraphPad Prism Version 6 (GraphPad Software, La Jolla, CA, USA) and IBM Statistical Package for Social Science version 21 (Armonk, NY: IBM Corp.). Normality of distribution was checked using the Shapiro-Wilk test. The data were expressed as mean ± standard deviation (SD) for normally distributed variables or median [1st−3rd quartiles] for non-normally distributed variables. The Chi-squared test was applied to evaluate nominal variables. To compare quantitative variables between groups the Student's *T*-test and the Mann-Whitney *U*-test were used, as appropriate. A multivariate logistic regression analysis with a forward conditional method was performed to evaluate the association between *patient tailored therapy* and ICU-mortality, adjusted for arbitrarily selected variables that were deemed to be relevant for the outcome. These included variables related to the type of infection (source of infection, multidrug-resistance), patient's severity on ICU-admission (APACHE II score), available data on comorbidities (chronic heart failure, diabetes mellitus), immunosuppression, indices of organ function (SOFA score, heart rate, mean arterial pressure, lactate levels). In order to obtain more robust evidence of the outcome, we also decided to perform a propensity score-matched analysis. A Propensity Score for the likelihood of being part of the group treated with the *patient tailored therapy* was obtained by means of multiple logistic regression. The following clinically relevant variables were included in the score: source of infection, multidrug-resistant pathogens infection, immunosuppression, SOFA score, heart rate, mean arterial pressure, lactate in the 1st h after initiation of norepinephrine infusion, APACHE II at ICU admission, comorbidities (chronic heart failure, diabetes mellitus). Matching was then performed in a 1:1 fashion with a caliper of 0.1 (11) in order to account for the different characteristics between the *patient tailored therapy* group and historical controls. Comparisons between propensity matched groups were made by means of paired *t*-test for normally distributed variables or Wilcoxon test for non-normally distributed variables and McNemar's test for proportions. In order to show the magnitude of differences between the two groups, we reported the correlation coefficient r as effect size for all comparisons: a value of ± 0.1 indicates a small effect, ± 0.3 a medium effect, ± 0.5 a large effect (12). A two-tailed *p* < 0.05 was used to define statistical significance.

## Results

**Figure 2:**
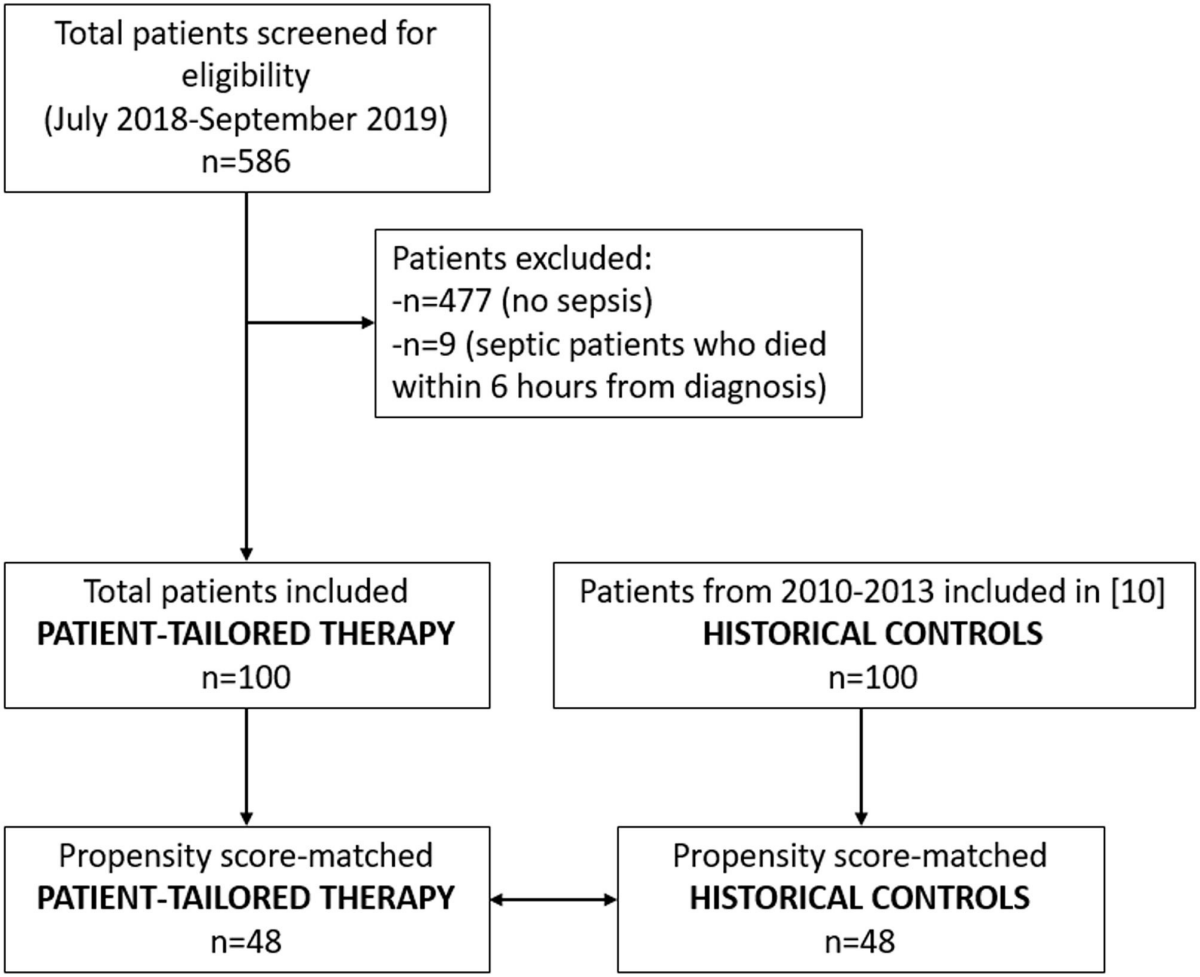
Study flow chart.

The study flow chart is reported in Figure 2. The full dataset of this study is available at https://doi.org/10.17026/dans-zyf-qvax.

General characteristics of the historical controls and patient-tailored therapy cohort are reported in Table 1. Patients in the patient-tailored therapy group had higher SAPS II on ICU-admission but lower SOFA score at the time of initiation of norepinephrine and the number of those with pre-existing immunosuppression tended to be lower in this group as compared to historical controls. Although the main source of infection was respiratory in both groups, among historical controls we observed a higher prevalence of abdominal sepsis while the patient-tailored therapy group showed more cases of genito-urinary sepsis and bactaeremia. The distribution of pathogens was similar between the 2010–2013 and the 2018–2019 cohorts (*p* = 0.227): gram-positive (21 vs. 28%), gram-negative (40 vs. 41%), virus (8 vs. 5%), fungi (6 vs. 4%), atypical (0 vs. 2%), unknown (31 vs. 20%). The cytokine profile in the patient-tailored therapy cohort was: IL-1beta 4 [4-9] pg/ml; IL-6 354 [507–1,980] pg/ml; IL-8 317 [119–2,439] pg/ml; IL-10 35 [11-365] pg/ml; TNF-alpha 34 [14-82] pg/ml.

**Table 1:** General characteristics of the two cohorts.

	**Unmatched entire cohort**				**Propensity-score matched cohort**			
	**Historical controls** **(*n* = 100)**	**Patient-tailored** **(*n* = 100)**	**Effect size** **(*r*)**	***p***	**Historical controls** **(*n* = 48)**	**Patient-tailored** **(*n* = 48)**	**Effect size** **(*r*)**	***p***
Age (years)	69 [55–75]	67 [49–76]	−0.046	0.517	72 [58–78]	60 [50–72]	0.229	0.027
Males	59	56	−0.030	0.668	31 (65%)	25 (52%)	−0.127	0.301
**Comorbidities (*****n*****, %)**								
*Chronic heart failure*	10	8	−0.035	0.806	3 (6%)	3 (6%)	0	0.999
*Diabetes mellitus*	24	21	−0.036	0.735	12 (25%)	12 (25%)	0	0.999
SAPS II (ICU admission)	57 ± 17	61 ± 16	0.141	0.047	59 ± 17	57 ± 15	0.051	0.731
APACHE II (ICU admission)	23 [19–30]	23 [19–28]	−0.033	0.641	24 ± 6	23 ± 6	0.179	0.228
SOFA score (1st h)	11 ± 3	10 ± 2	0.193	0.007	11 ± 3	11 ± 3	0.080	0.609
MAP (mmHg) 1st h	76 ± 13	75 ± 15	0.026	0.719	75 ± 15	78 ± 16	0.140	0.338
Norepinephrine (mcg/kg/min) 1st h	0.40 [0.16–0.77]	0.30 [0.16–0.50]	−0.116	0.101	0.39 [0.14–0.77]	0.28 [0.13–0.49]	0.118	0.252
Lactate (mmol/L) 1st h	2.4 [1.1–4.2]	2.3 [1.5–3.4]	−0.007	0.919	2.4 [1.1–5.2]	2.3 [1.5–4.7]	0.086	0.406
HR (bpm) 1st h	100 ± 22	98 ± 19	0.056	0.430	99 ± 24	99 ± 20	0.014	0.926
Immunosuppression	18	9	−0.132	0.063	6 (12%)	7 (15%)	0.030	0.999
Source of infection (*n*, %)			0.199	<0.001			0.039	0.906
*Respiratory*	51	49			24 (50%)	25 (52%)		
*Abdominal*	35	11			13 (27%)	11 (23%)		
*Genito-urinary*	2	12			2 (4%)	3 (6%)		
*Bacteraemia*	2	9			2 (4%)	1 (2%)		
*Skin and soft tissue*	7	7			5 (10%)	6 (12%)		
*Other*	1	2			0 (0%)	1 (2%)		
*Unknown*	2	10			2 (4%)	1 (2%)		
MDR infection	33	24	−0.100	0.110	16 (33%)	14 (29%)	0.045	0.826
ICU LOS (days)	11 [3-22]	12 [7-22]	−0.070	0.324	11 (4-34)	11 (7-24)	0.046	0.660
ICU Non-survivors (*n*, %)	57	32	−0.251	<0.001	29 (60%)	19 (40%)	−0.208	0.037
Hospital Non-survivors (*n*, %)	63	36	−0.270	<0.001	33 (69%)	19 (40%)	−0.293	0.007

*Propensity score analysis: patients were matched for source of infection, multi-drug resistant pathogen, pre-existing immunosuppression, pre-existing chronic heart failure, diabetes mellitus, APACHE II on ICU-admission, SOFA, HR, MAP, Lactate at the 1st hour of sepsis diagnosis*.

*For the effect size r: a value of ± 0.1 indicates a small effect, ± 0.3 a medium effect, ± 0.5 a large effect*.

*SAPS II, Simplified Acute Physiology Score II; APACHE II, Acute Physiology and Chronic Health Evaluation II; SOFA, Sequential Organ Failure Assessment; MAP, mean arterial pressure; HR, heart rate; MDR, multi-drug resistant; ICU LOS, length of stay in the Intensive Care Unit*.

Patients in the patient-tailored therapy group received a lower amount of fluids in the 1st 24 h of norepinephrine infusion and a lower dose of norepinephrine (Table 2). A higher number of patients underwent renal replacement therapy in the 1st 24 h as compared to historical controls. The use of hemodynamic monitoring in the 1st 24 h of norepinephrine administration was greatly increased in our ICU in 2018–2019 in comparison to 2010–2013. A substantial number of patients in the patient-tailored therapy group received albumin, pentaglobin and blood purification therapies, while these treatments were not used in the period 2010–2013 (Table 2). The use of steroids as adjunctive therapy for sepsis was similar in the two time periods.

**Table 2:** Comparison of treatments between the two study cohorts.

	**Unmatched entire cohort**				**Propensity-score matched cohort**			
	**Historical controls (*n* = 100)**	**Patient-tailored (*n* = 100)**	**Effect size (*r*)**	***p***	**Historical controls (*n* = 48)**	**Patient-tailored (*n* = 48)**	**Effect size (*r*)**	***p***
RRT (1st 24 h)	10	25	0.197	0.005	6 (12%)	15 (31%)	0.227	0.047
Mechanical Ventilation (1st 24 h)	99	98	−0.041	0.561	48 (100%)	48 (100%)	0	0.999
Fluid balance 1st 24 h*	513 [−96, 2,351] (*n* = 88)	347 [−854, 1,720] (*n* =93)	−0.107	0.150	430 [−90, 2,500] (*n* = 41)	182 [−634, 1,725] (*n* = 41)	0.098	0.395
Total fluid 1st 24 h (ml/kg)*	59 [47–79] (*n* = 88)	50 [38–71] (*n* = 93)	−0.164	0.027	54 [45–72] (*n* = 41)	47 [35–69] (*n* = 41)	0.128	0.264
Norepinephrine MAX 1st 24 h (mcg/kg/min)	0.61 [0.27–1.02]	0.40 [0.21–0.71]	−0.211	0.003	0.66 [0.19–1.04]	0.42 [0.20–0.67]	0.248	0.014
**Other vasoactive/inotropic agents (1st 24 h)**								
*Dobutamine*	20	24	0.048	0.495	9 (19%)	9 (19%)	0	0.999
*Dopamine*	9	3	−0.126	0.074	2 (4%)	1 (2%)	−0.060	0.999
*Levosimendan*	4	10	0.118	0.096	2 (4%)	3 (6%)	0.047	0.999
*Terlipressin*	0	9	0.217	0.002	0 (0%)	3 (6%)	0.180	0.242
Pentaglobin, n	0	18	0.314	<0.001	0 (0%)	11 (23%)	0.360	0.001
Cytosorb, n	0	15	0.285	<0.001	0 (0%)	8 (17%)	0.301	0.006
Toraymyxin, n	0	5	0.160	0.059	0 (0%)	3 (6%)	0.180	0.242
Albumin, n	0	71	0.742	<0.001	0 (0%)	35 (73%)	0.757	<0.001
Steroids, n	14	9	−0.078	0.376	7 (15%)	4 (8%)	−0.098	0.523
Hemodynamic monitoring** (1st 24 h), n	10	63	0.550	<0.001	5 (10%)	31 (65%)	0.559	<0.001

*Patients were matched for source of infection, multi-drug resistant pathogen, pre-existing immunosuppression, SAPS II on ICU-admission, SOFA, HR, MAP, Lactate at the 1st hour of sepsis diagnosis*.

*In order to show the magnitude of differences between the two groups, we reported the effect size r: a value of ± 0.1 indicates a small effect, ± 0.3 a medium effect, ± 0.5 a large effect*.

*RRT, renal replacement therapy*.

* *Fluid balance and total fluid intake in the 1st 24 h are calculated only for patients surviving for more than 24 h (number of patients is shown in parenthesis)*.

** *This includes invasive or mini-invasive measurement of cardiac output by means of either trans-pulmonary thermodilution or pulse contour analysis*.

ICU-mortality was lower in the patient-tailored therapy cohort as compared to historical controls (32 vs. 57%, *p* < 0.001). The main causes of death were multi-organ failure (79% in historical controls, 75% in the patient-tailored therapy cohort), respiratory failure (14 and 12%, respectively), and intractable hypotension (7 and 6%, respectively). Patient-tailored therapy was associated with a lower risk of ICU-mortality even after adjusting for source of infection, presence of multi-drug resistant pathogen, pre-existing immunosuppression, SOFA, heart rate, mean arterial pressure, lactate levels at the 1st h of norepinephrine infusion, APACHE II score on ICU-admission, pre-existing chronic heart failure and diabetes mellitus (Table 3).

**Table 3:** Multivariate binary logistic regression for ICU-mortality.

**Variable**	**Odds ratio [95% CI]**	***p***
Patient-tailored therapy	0.331 [0.166–0.658]	0.002
SOFA (1st h)	1.238 [1.077–1.423]	0.003
Immunosuppression	3.297 [1.106–9.825]	0.032
MAP (1st h)	0.968 [0.943–0.993]	0.014
Lactate levels (1st h)	1.239 [1.095–1.401]	0.001

*Variables included in the model were source of infection, multidrug-resistant pathogens infection, immunosuppression, SOFA score (1st h of norepinephrine infusion), heart rate, mean arterial pressure, lactate levels, APACHE II on admission, pre-existing chronic heart failure and diabetes mellitus. Forward conditional method*.

*MAP, mean arterial pressure; APACHE II, Acute Physiology and Chronic Health Evaluation Score II*.

After propensity score matching, we selected 48 patients in historical control group and 48 patients in the patient-tailored therapy cohort with similar general characteristics (Table 1). ICU-mortality was lower in the patient-tailored therapy matched subgroup as compared to the historical control subgroup (40 vs. 60%, *p* = 0.037). A comparison of treatments in the two propensity matched groups is shown in Table 2.

### Patients With Septic Shock

A total of 56 patients among historical controls and 58 patients in the patient-tailored therapy cohort had high lactate levels, meeting the criteria for septic shock according to the Sepsis-3 definitions (Supplementary File 1).

Among these patients, ICU-mortality was 43.1% in the patient-tailored therapy cohort and 73.2% in the standard therapy cohort (*p* = 0.001). Patient-tailored therapy was associated with lower ICU-mortality independent of the source of infection, presence of multi-drug resistant pathogen, pre-existing immunosuppression, SOFA at time of initiation of norepinephrine, heart rate, mean arterial pressure, lactate levels, pre-existing chronic heart failure, diabetes mellitus and APACHE on ICU-admission (adjusted odds ratio 0.269 [95% CI 0.112–0.644], *p* = 0.003, Supplementary File 2). After propensity score matching (for source of infection, multi-drug resistant pathogen, pre-existing immunosuppression, APACHE on ICU-admission, chronic heart failure, diabetes mellitus, SOFA, HR, MAP, lactate levels and initial dose of norepinephrine), ICU-mortality was 47% in the patient-tailored therapy group (*n* = 30) and 77% in the historical control group (*n* = 30) (*p* = 0.039, Supplementary File 1).

### Adherence to the Patient-Tailored Therapy Protocol

In the patient-tailored therapy cohort, five patients did not receive Pentaglobin infusion despite IgM plasma levels <60 mg/dl. Hemoadsorption was not applied in 5 patients who met the criteria for blood purification therapy. In 17 cases, albumin was not administered although plasma levels were <2.5 g/dl. Five patients requiring a norepinephrine dosage > 1 mcg/kg/min did not receive terlipressin infusion.

## Discussion

In this single-center retrospective observational study, patients with norepinephrine-dependent sepsis admitted to our ICU after the institution of a patient-tailored protocol showed significantly lower mortality in comparison to a group of patients admitted between 2010 and 2013. The association between patient-tailored therapy and reduced mortality remained after adjusting for the main clinical severity parameters or restricting the analysis to patients who met the Sepsis-3 definitions for septic shock (1). Nonetheless, we must recognize that such association does not imply a cause-effect relationship. The outcome of septic patients may have changed over the last years for many other factors unrelated to this personalized approach, which need to be discussed.

The therapeutic approach to septic patients has changed a lot in our ICU in the last years. The approach to hemodynamic stabilization, as regards both fluid therapy and vasoactive agents, has changed. The reduction in norepinephrine dosage in the 1st 24 h and the increase in the use of terlipressin, together with the lower fluid intake in the 1st 24 h, suggest that we now tend to avoid an excessive fluid administration and limit the use of excessive doses of norepinephrine, in favor of an earlier association with other vasoactive agents. Moreover, the widespread use of hemodynamic monitoring in 2018–2019 as compared to 2010–2013 likely reflects a different approach to the hemodynamic stabilization of septic patients, with the implementation of a fluid resuscitation guided by a more thorough hemodynamic assessment, which may have contributed to limit the amount of fluids administered and the vasopressor dosage. In addition, a higher number of patients currently receive RRT in the 1st 24 h. A restrictive fluid administration strategy, allowed by an earlier initiation of RRT and more rational use of vasoactive and inotropic agents, could have a beneficial effect on the outcome since many recent studies demonstrated that fluid overload can lead to several complications such as pulmonary oedema, congestive heart failure, delayed wound healing and impaired bowel function (13).

At present, all septic patients in our ICU with low serum albumin levels receive human serum albumin, while this was not used in 2010–2013. This practice was implemented in our unit after the publication of the results of the ALBIOS trial (14) that showed significant hemodynamic advantages with albumin administration and a possible reduction in mortality in the subgroup of patients with septic shock. The use of albumin in our patient-tailored therapy cohort may have contributed to the reduction in norepinephrine dosage.

Different therapies, such as immunomodulation and extracorporeal removal strategies, has been introduced. Despite absence of clear recommendations for the use of immunoglobulin preparations and blood purification techniques in *Surviving Sepsis Campaign* (8), a number of clinical studies has shown a beneficial effect of IgM-enriched immunoglobulin solutions and blood purification with Cytosorb adsorber during sepsis. In a meta-analysis of RCTs, we previously showed that the infusion of immunoglobulins may reduce mortality in septic patients, although the overall quality of the available evidence remains low (15). In a recent randomized controlled study by our group, IgM-enriched immunoglobulins were able to improve sublingual microvascular perfusion during sepsis (16). In patients with septic shock requiring RRT, we showed that extracorporeal blood purification with Cytosorb adsorber was associated with an improvement in sublingual microcirculation (17). It is known that during sepsis a macro-hemodynamic improvement may not be accompanied by a parallel restoration of microvascular perfusion (18). Massive cytokine release in sepsis leads to leukocyte activation, oxidative stress, endothelial glycocalyx dysfunction and impaired nitric oxide pathway, resulting in impaired hemorheology, loss of microvascular tone, microcirculatory shunting, tissue oedema and oxygen extraction deficit (17). The implementation of cytokine removal strategies, by acting on the main pathophysiological mechanism of microcirculatory dysfunction, may have a beneficial impact on tissue perfusion and organ function. It is reasonable to think that these treatments could show the best impact on outcome if administered to selected patients (e.g., those with lower plasma IgM levels or more severe inflammatory response) following an individualized, patient-tailored approach. Of note, we used the Cytosorb cartridge in combination with RRT as a dedicated extracorporeal circuit is not available in our ICU. However, RRT is generally not a pre-requisite for hemoadsorption/hemoperfusion therapies: their use as stand-alone therapies with dedicated extracorporeal circuits could allow an earlier implementation of blood purification, with a potentially better impact on outcome.

Our data are in line with the current evidence. Due to the extreme heterogeneity of septic patients (2), most trials failed to show a mortality benefit from treatments including immunoglobulins or blood purification techniques (19–22). For this reason, an individualized approach is most frequently required, that will take into account the basal patient characteristics, underlying infection, organ dysfunction severity and the individual immune response (5). By performing a Latent class analysis of the PROWESS Shock study, Gardlung et al. highlighted that septic shock is a complex entity characterized by different phenotypes, and each of these phenotypes would benefit from targeted therapies based on patient characteristics (23). Consistently, Zhang et al. identified four subclasses of septic patients that showed different responses to fluid resuscitation with different outcomes (24). Of note, in the present study, the adjunctive treatments, if individually analyzed, do not show this positive correlation with survival. This is in line with the concept of “bundles of care” that was endorsed by the Surviving Sepsis Campaign based on the assumption that the association of different treatments will be able to provide the best impact on outcome (25).

Our study has several limitations. First of all, due to its retrospective design and the use of an historical control group, this study does not allow to discriminate the potential benefit of patient-tailored therapy from the impact of other practice changes that occurred from 2010 to 2019. We cannot exclude that the observed difference in mortality was determined by factors not exclusively related to the individualized therapeutic approach or was influenced by therapeutic elements that we may have missed to consider. Among these, the type of fluids infused: it is likely that a lower amount of synthetic colloids was used in the 2018–2019 cohort, according to the most recent recommendations of the SSC guidelines (8) after the publication of RCTs showing a higher risk of mortality and acute kidney injury with hydroxyethyl starches (26). Unfortunately, we do not have access to data on the specific type of fluids (crystalloids or colloids) for the 2010–2013 cohort. Similarly, data on antimicrobials were not available. Even if the distribution of pathogens was similar between the two cohorts, changes in antimicrobial strategies over years could have played a relevant role in determining patient outcome.

This study cannot demonstrate any cause-effect relationship between the individualized approach and patients' outcome, but can only suggest associations. Even if we made all efforts to adjust for the main clinical severity indices, unmeasured confounders may still bias our results. We used a group of historical controls, consisting of patients admitted to our ICU in the period between 2010 and 2013, for which some information of therapeutic elements were no longer available. Differences in the base populations from which the patients were selected may limit the comparability between the two cohorts. While at present (as well as in the period 2018–2019) our unit is an exclusively medical and trauma ICU, in the period 2010–2013 also post-operative patients were admitted. This may at least partly explain the higher prevalence of abdominal sepsis cases in the standard therapy group. Moreover, the SOFA score was slightly lower in the 2018–2019 cohort. A general improvement in the care of septic patients including an earlier recognition of this condition, also related to the diffusion of the use of *quick SOFA* (1) outside the ICU, may justify such difference and could have contributed to mortality reduction. These potential confounding factors were included in multivariate logistic regression and propensity score analyses. The prevalence of comorbidities in the two cohorts could not be analyzed in details, as only incomplete information of the patients' medical history was available for the 2010–2013 dataset. Unfortunately, we could not collect data from a more recent control group of patients, as our electronic medical reports are fully available and easy accessible only for 3 years before being archived.

Finally, the sample size was arbitrarily chosen based on the size of the old sample available. Even if the study is retrospective and observational, the lack of protocol registration to any public register does not allow verification of design and statistical analysis.

## Conclusion

In a group of 100 patients with norepinephrine-dependent sepsis admitted to our ICU after the institution of a protocol of patient-tailored therapy, we found a significantly lower mortality in comparison to a group of historical controls. This study cannot demonstrate a clear effect of the *patient tailored therapy* on the outcome. Nevertheless, our results encourage the design of future studies specifically aimed to test the impact of an individualized therapeutic approach on the outcome of septic patients.

## Data Availability Statement

The datasets generated for this study can be found in online repositories. The names of the repository/repositories and accession number(s) can be found at: https://doi.org/10.17026/dans-zyf-qvax.

## Ethics Statement

The studies involving human participants were reviewed and approved by Comitato Etico Regionale delle Marche. Written informed consent for participation was not required for this study in accordance with the national legislation and the institutional requirements.

## Author Contributions

ED and EC collected the data, performed the statistical analysis, interpreted the data, and drafted the manuscript. RD, AC, CS, SB, DDF, SP, SV, and AD participated in the collection, analysis of the data, and interpretation of the results. EA designed the study and revised the manuscript. AD designed the study, participated in the statistical analysis, interpretation of the data, and revised the manuscript. All authors approved the submitted version of the manuscript and agreed both to be personally accountable for the author's own contributions and to ensure that questions related to the accuracy or integrity of any part of the work, even ones in which the author was not personally involved, are appropriately investigated, resolved, and the resolution documented in the literature. All authors read and approved the final manuscript.

## Conflict of Interest

The authors declare that the research was conducted in the absence of any commercial or financial relationships that could be construed as a potential conflict of interest.
