# Supplementary material for: Variation in the Outcome of Norepinephrine-Dependent Septic Patients After the Institution of a Patient-Tailored Therapy Protocol in an Italian Intensive Care Unit: Retrospective Observational Study
Source: Front Med (Lausanne). 2020 Nov 5;7:592282. doi: 10.3389/fmed.2020.592282 (PMC7674935; doi:10.3389/fmed.2020.592282)
Supplement: Supplementary file 1 [file Data_Sheet_1.docx]

**Supplementary Material 1 – Comparison between historical controls and patient-tailored therapy restricted to patients with septic shock according to Sepsis-3 definitions.**

|  | **Unmatched entire cohort** | | | | **Propensity-score matched cohort** | | | |
| --- | --- | --- | --- | --- | --- | --- | --- | --- |
|  | **Standard therapy (n=56)** | **Patient-tailored therapy (n=58)** | **Effect size (r)** | **p** | **Standard therapy (n=30)** | **Patient-tailored therapy (n=30)** | **Effect size (r)** | **p** |
| Age (years) | 71 [59-77] | 63 [49-75] | -0.135 | 0.151 | 69 [58-75] | 58 [49-74] | 0.228 | 0.079 |
| Males | 29 (51.8%) | 32 (55.2%) | 0.034 | 0.851 | 17 (57%) | 16 (53%) | -0.033 | 0.999 |
| Comorbidities (n, %) |  |  |  |  |  |  |  |  |
| *Chronic heart failure* | 7 (12%) | 5 (9%) | -0.063 | 0.554 | 5 (17%) | 2 (7%) | -0.156 | 0.424 |
| *Diabetes mellitus* | 14 (25%) | 8 (14%) | -0.142 | 0.158 | 7 (23%) | 3 (10%) | -0.179 | 0.299 |
| SAPS II (ICU admission) | 61 [47-77] | 64 [53-76] | -0.044 | 0.644 | 62±17 | 64±16 | 0.082 | 0.660 |
| APACHE II (ICU admission) | 26 [20-32] | 23 [20-29] | -0.113 | 0.230 | 27±7 | 24±5 | 0.320 | 0.079 |
| SOFA score (1st h sepsis) | 12±3 | 11±2 | 0.265 | 0.006 | 12±3 | 12±3 | 0.089 | 0.632 |
| MAP (mmHg) 1st h sepsis | 73 [64-85] | 73 [61-87] | -0.025 | 0.790 | 73 [64-84] | 72 [57-83] | 0.085 | 0.519 |
| Norepinephrine (mcg/kg/min) 1st h | 0.500 [0.202-0.832] | 0.400 [0.195-0.722] | -0.105 | 0.264 | 0.460 [0.140-0.890] | 0.500 [0.195-0.850] | -0.028 | 0.848 |
| Lactate (mmol/L) 1st h | 3.8 [2.7-7.1] | 3 [2.5-6.8] | -0.117 | 0.211 | 4.1 [2.4-7.1] | 3.4 [2.4-10.4] | -0.034 | 0.810 |
| HR (bpm) 1st h | 100 [88-125] | 99 [86-120] | -0.066 | 0.481 | 98 [86-128] | 106 [87-124] | -0.036 | 0.786 |
| Immunosuppresion | 14 (25%) | 7 (12.1%) | -0.167 | 0.093 | 8 (27%) | 4 (13%) | 0.167 | 0.333 |
| Source of infection |  |  | 0.107 | 0.004 |  |  | 0.000 | 0.791 |
| *respiratory* | 27 (48.2%) | 28 (48.3%) |  |  | 18 (60%) | 18 (60%) |  |  |
| *abdominal* | 22 (39.3%) | 8 (13.8%) |  |  | 7 (23%) | 8 (27%) |  |  |
| *genito-urinary* | 0 (0%) | 10 (17.2%) |  |  | 0 (0%) | 0 (0%) |  |  |
| *bacteraemia* | 2 (3.6%) | 7 (12.1%) |  |  | 2 (7%) | 1 (3%) |  |  |
| *skin and soft tissue* | 2 (3.6%) | 2 (3.4%) |  |  | 2 (7%) | 2 (7%) |  |  |
| *other* | 1 (1.8%) | 1 (1.7%) |  |  | 1 (3%) | 0 (0%) |  |  |
| *unknown* | 2 (3.6%) | 2 (3.4%) |  |  | 0 (0%) | 1 (3%) |  |  |
| MDR infection | 16 (28.6%) | 13 (22.4%) | -0.071 | 0.521 | 8 (27%) | 4 (13%) | 0.167 | 0.333 |
| ICU LOS (days) | 7 [2-20] | 10 [5-21] | -0.167 | 0.112 | 4 [1-20] | 11 [3-26] | -0.106 | 0.420 |
| ICU Non-survivors | 41 (73.2%) | 25 (43.1%) | -0.305 | 0.001 | 23 (77%) | 14 (47%) | -0.308 | 0.039 |
| Hospital Non-survivors | 41 (73.2%) | 26 (44.8%) | -0.288 | 0.002 | 23 (77%) | 14 (47%) | -0.308 | 0.039 |
| RRT (1st 24h) | 11 (19.6%) | 18 (31%) | 0.131 | 0.199 | 5 (17%) | 10 (33%) | 0.192 | 0.233 |
| Mechanical Ventilation (1st 24h) | 56 (100%) | 58 (100%) | - | - | 30 (100%) | 30 (100%) | - | - |
| Fluid balance 1st 24h* | 1509 [-2.75, 2846] (45) | 686 [-855, 2126] (52) | -0.113 | 0.296 | 1509 [-133, 2858] (22) | 1507 [-855, 3007] | 0.012 | 0.949 |
| Total fluid 1st 24h (ml/kg)* | 68±27 (45) | 66±34 (52) | -0.087 | 0.820 | 71±24 (22) | 71±40 (22) | -0.135 | 0.388 |
| Norepinephrine MAX 1st 24h (mcg/kg/min) | 0.868 [0.514-1.200] | 0.550 [0.365-0.800] | -0.277 | 0.003 | 0.757 [0.433-1.205] | 0.735 [0.475-1.000] | 0.085 | 0.522 |
| Other vasoactive agents (1st 24h) |  |  |  |  |  |  |  |  |
| *dobutamine* | 10 (17.9%) | 18 (31%) | 0.153 | 0.129 | 8 (27%) | 9 (30%) | 0.037 | 0.999 |
| *dopamine* | 4 (7.1%) | 2 (3.4%) | -0.083 | 0.434 | 1 (3%) | 1 (3%) | 0.000 | 0.999 |
| *levosimendan* | 2 (3.6%) | 9 (15.5%) | 0.202 | 0.053 | 2 (7%) | 4 (13%) | 0.111 | 0.671 |
| *terlipressin* | 0 (0%) | 8 (13.8%) | 0.270 | 0.006 | 0 (0%) | 5 (17%) | 0.301 | 0.052 |
| Pentaglobin, n | 0 (0%) | 17 (29.3%) | 0.411 | <0.001 | 0 (0%) | 9 (30%) | 0.420 | 0.002 |
| Cytosorb, n | 0 (0%) | 11 (19%) | 0.321 | 0.001 | 0 (0%) | 8 (27%) | 0.392 | 0.005 |
| Toraymyxin, n | 0 (0%) | 4 (6.9%) | 0.187 | 0.119 | 0 (0%) | 3 (10%) | 0.229 | 0.237 |
| Albumin, n | 0 (0%) | 42 (72.4%) | 0.750 | <0.001 | 0 (0%) | 22 (73%) | 0.761 | <0.001 |
| Steroids, n | 7 (12%) | 9 (15%) | 0.043 | 0.789 | 4 (13%) | 5 (17%) | 0.047 | 0.999 |
| Hemodynamic monitoring, n | 7 (12%) | 44 (76%) | 0.637 | <0.001 | 2 (7%) | 23 (77%) | 0.710 | <0.001 |

Patients were matched for source of infection, multi-drug resistant pathogen, pre-existing immunosuppression, APACHE II on ICU-admission, comorbidities (chronic heart failure, diabetes mellitus), SOFA, HR, MAP, dose of norepinephrine and lactate levels at the first hour of septic shock.

In order to show the magnitude of differences between the two groups, we reported the effect size *r*: a value of ±0.1 indicates a small effect, ±0.3 a medium effect, ±0.5 a large effect.

*SAPS II* Simplified Acute Physiology Score II, *APACHE II* Acute Physiology and Chronic Health Evaluation II, *SOFA* Sequential Organ Failure Assessment, *MAP* mean arterial pressure, *HR* heart rate, *MDR* multi-drug resistant, *ICU LOS* length of stay in the Intensive Care Unit

*Fluid balance and total fluid intake in the first 24 hours are calculated only for patients surviving for more than 24h (number of patients is shown in parenthesis).

**Supplementary Material 2 -** **Multivariate binary logistic regression for ICU-mortality restricted to patients with septic shock according to Sepsis-3 definitions.**

Variables included in the model were source of infection, multidrug-resistant pathogens infection, immunosuppression, SOFA score, heart rate, mean arterial pressure, lactate levels (in the first hour after sepsis diagnosis), APACHE II on admission, chronic heart failure, diabetes mellitus. Forward conditional method.

| **Variable** | **Odds ratio [95% CI]** | **p** |
| --- | --- | --- |
| Patient-tailored therapy | 0.269 [0.112-0.644] | 0.003 |
| Immunosuppression | 5.621 [1.412-22.377] | 0.014 |
| Lactate levels (first hour) | 1.273 [1.102-1.469] | 0.001 |
